# Susceptibility (risk and protective) factors for in-patient violence and self-harm: prospective study of structured professional judgement instruments START and SAPROF, DUNDRUM-3 and DUNDRUM-4 in forensic mental health services

**DOI:** 10.1186/1471-244X-13-197

**Published:** 2013-07-27

**Authors:** Zareena Abidin, Mary Davoren, Leena Naughton, Olivia Gibbons, Andrea Nulty, Harry G Kennedy

**Affiliations:** 1 National Forensic Mental Health Service, Central Mental Hospital, Dublin 14, Dundrum, Ireland; 2 Department of Psychiatry, Trinity College, Dublin, Ireland

**Keywords:** Risk, Violence, Self-harm, SAPROF, START, DUNDRUM toolkit, HCR-20, S-RAMM, Forensic, Protective

## Abstract

**Background:**

The START and SAPROF are newly developed fourth generation structured professional judgement instruments assessing strengths and protective factors. The DUNDRUM-3 and DUNDRUM-4 also measure positive factors, programme completion and recovery in forensic settings.

**Methods:**

We compared these instruments with other validated risk instruments (HCR-20, S-RAMM), a measure of psychopathology (PANSS) and global function (GAF). We prospectively tested whether any of these instruments predict violence or self harm in a secure hospital setting (n = 98) and whether they had true protective effects, interacting with and off-setting risk measures.

**Results:**

SAPROF and START-strengths had strong inverse (negative) correlations with the HCR-20 and S-RAMM. SAPROF correlated strongly with GAF (r = 0.745). In the prospective in-patient study, SAPROF predicted absence of violence, AUC = 0.847 and absence of self-harm AUC = 0.766. START-strengths predicted absence of violence AUC = 0.776, but did not predict absence of self-harm AUC = 0.644. The DUNDRUM-3 programme completion and DUNDRUM-4 recovery scales also predicted in-patient violence (AUC 0.832 and 0.728 respectively), and both predicted in-patient self-harm (AUC 0.750 and 0.713 respectively). When adjusted for the HCR-20 total score however, SAPROF, START-S, DUNDRUM-3 and DUNDRUM-4 scores were not significantly different for those who were violent or for those who self harmed. The SAPROF had a significant interactive effect with the HCR-dynamic score. Item to outcome studies often showed a range of strengths of association with outcomes, which may be specific to the in-patient setting and patient group studied.

**Conclusions:**

The START and SAPROF, DUNDRUM-3 and DUNDRUM-4 can be used to assess both reduced and increased risk of violence and self-harm in mentally ill in-patients in a secure setting. They were not consistently better than the GAF, HCR-20, S-RAMM, or PANSS when predicting adverse events. Only the SAPROF had an interactive effect with the HCR-20 risk assessment indicating a true protective effect but as structured professional judgement instruments all have additional content (items) complementary to existing risk assessments, useful for planning treatment and risk management.

## Background

The assessment of risk of violence [1-4] has developed into ‘structured professional judgement’ approaches to risk assessment [1,5,6]. Identifying risk factors is held to be an aid to treatment planning [7] and perhaps for this reason risk assessment has come to pervade forensic mental health practice.

Doyle and Dolan [8] reviewed what they called ‘generational’ developments or phases in risk assessment. The ‘first generation’ - unstructured clinical or professional judgement [9] gave way to the second generation actuarial risk assessment tools [8]. However, the actuarial approach was criticised for focusing on a limited number of factors without taking into account potentially crucial case-specific idiosyncratic factors [8,10]. A combination of both the clinical and actuarial approaches was required. This led to the development of the third generation risk assessment [8] described as empirically validated structured decision making [11] or structured professional judgement (SPJ) [12]. The leading structured professional judgement instrument for the assessment of risk of violence has been the Historical-Clinical-Risk Management-20 (HCR-20) [13]. This added the distinction between fixed historical risk factors and dynamic factors that are subject to change over time and in response to treatment. Although rated according to a set of defined risk items, the final judgement of risk level allows for clinical judgement rather than a simple actuarial score.

Forensic hospital patients and others like them are also at a greatly increased risk of suicide both in hospital and on returning to the community [14]. There has also been a recent interest in the assessment of risk of suicide and self-harm using structured professional judgement instruments [15].

Gaps in the structured professional judgement approach to risk assessment could be identified. Protective or resilience factors that might reduce risk of violence were first used in the structured clinical risk assessment instruments devised for children and adolescents [16,17]. This reflects not only the importance of resilience as a developmental factor in young people, but also the reality that protective factors are taken into account by clinicians when making decisions about risk and treatment. The assessment system for adults should therefore allow for a broader assessment of susceptibility factors – negative risk or vulnerability factors that increase the probability of violence and self harm, and positive, protective or resilience factors that reduce the risk of violence and self-harm. Several new SPJ risk assessment instruments have appeared that are designed to assess protective factors or progress in treatment and recovery as part of the assessment and management of susceptibility (risk and protective factors) for violence and self-harm.

The Short-Term Assessment of Risk and Treatability (START) is a clinical guide for the dynamic assessment of risks, strengths and treatability which is relevant to everyday psychiatric clinical practice [18]. According to the authors and others, the START is intended to “stimulate discussion about strengths, vulnerabilities and appropriate interventions and management” [19,20].

The SAPROF [21] is a recently-developed instrument for the assessment of factors protecting against violent acts. By specifically focusing on protective factors, the SAPROF aims to provide a more accurate and well-rounded assessment of risk for future violent behaviour [21].

The DUNDRUM-3 programme completion scale and DUNDRUM-4 recovery scale [22,23] are two structured professional judgement instruments designed for use as measures of progress along the recovery pathway for those detained in secure forensic psychiatric services. These have been shown to predict moves from more secure to less secure places, along with measures of risk [24] and they have been shown to predict conditional discharge from hospital to the community [25]. The DUNDRUM-1 triage security instrument is a measure of the need for therapeutic security and is designed to be a static measure of a quality that is complementary to and distinct from risk of violence [26]. It has been shown to influence moves between levels of therapeutic security [24] and it is used also as a benchmark to enable comparisons between studies [26]. The SPJ instruments of the DUNDRUM toolkit are all designed to be complimentary to measures of risk of violence or self-harm. The DUNDRUM-3 and DUNDRUM-4 are included here as they can be conceptualised as positive or protective factors likely to reduce the risk of violence and self-harm.

Rutter [27] pointed out that a protective or resilience factor should do more than simply predict the absence of harm or adverse outcomes, since predicting the absence of harm is merely the absence of risk. Risk or vulnerability factors (or their reciprocals, measuring the absence of risk) and protective or resilience factors can be validated as predictive or not using the receiver operating characteristic, as a means of taking into account base rate variations between samples [28]. The strength of association in the specific population and setting studied can be assessed with unadjusted odds ratios. According to Rutter [27] a truly protective factor would interact with risk factors to reduce the probability of an adverse event or outcome, even when risk factors were present. This requires a form of analysis of interactive effects additional to that normally used to validate risk factors.

### Objectives

In this prospective study we set out to assess psychometric properties, concurrent validity and criterion outcome measures of the validity of the START and SAPROF. We prospectively tested whether START and SAPROF, DUNDRUM-3 and DUNDRUM-4 would predict adverse events (or the absence of adverse events), violence or self harm. We compared these to existing validated instruments for the assessment of risk of violence (HCR-20) and self-harm (S-RAMM) and examined whether they accounted for any element of statistical prediction over and above an existing ‘gold standard’ instrument for the assessment of risk of violence, the HCR-20. We also examined the predictive properties of measures of symptoms (PANSS [29,30]) and global function (GAF [31]) treating these as another standard to be beaten – are specific risk assessment instruments and their constituent items better than assessing symptoms and function?

## Method

### Study design

This is a naturalistic six month prospective cohort study of in-patients in a therapeutically secure forensic hospital. Data were gathered as part of the clinical audit of service delivery and the study was approved by the research ethics, audit and effectiveness committee of the National Forensic Mental Health Service. All patients gave informed consent to participate.

### Setting

The Central Mental Hospital is a 94 bed forensic secure hospital providing high, medium and low security integrated on a single campus. The hospital is the only legally designated centre for forensic mental health treatments for a population of 4.6 million. At the time of the study the hospital was organised into a series of eight units from high secure admission and intensive care through medium secure and low secure to pre-discharge and community high support places so that the location at the start of the study period can be used as an index of the level of therapeutic security for the environment in which the patient is located [24].

### Participants

All patients at the Central Mental Hospital during the period March to April 2010 (n = 100) with severe mental illness participated as part of routine assessments of risk and outcome measures.

### Variables and data sources

The researchers who made ratings or collated them were each blind to the work of the others. One post membership psychiatric trainee (ZA) rated the START and SAPROF by interviewing patients, reviewing case notes and speaking to members of the multi-disciplinary team and ward-based nursing staff. The START takes a list of risk factors and treats each one as both a risk factor and a protective factor. The SAPROF includes items thought to be protective against violence such as intelligence, secure attachment in childhood and empathy that are not included in existing risk assessment instruments. Two post membership psychiatric trainees (LN and OG) carried out interviews using the PANSS and GAF.

The HCR-20 provides ratings for ten stable historical risk factors (HCR-H items) though we omitted item H7 ‘psychopathy’ as this was not in routine use, five current ‘clinical’ (HCR-C) and five future ‘risk management’ (HCR-R) items. Each item is scored 0 to 2 and the total scale is scored 0 to 38. The ‘C’ and ‘R’ items added together constitute a ‘dynamic’ or change sensitive score (HCR-dynamic, scored 0 to 20). Similarly, the Suicide Risk Assessment and Management Manual S-RAMM [15] is made up of 23 items each scored 0 to 2 with an overall scale score from 0 to 46 and generates a nine item stable, background score (S-RAMM-B) and change sensitive dynamic scales for eight current (S-RAMM-C) and five future (S-RAMM-F) risk items for self-harm or suicide, the latter two of which combine as a thirteen item dynamic score (S-RAMM-dynamic) rated 0 to 26. The HCR-20 and S-RAMM scales were collated from team assessments by an advanced nurse practitioner (AN) who ensured quality and fidelity to the handbook definitions.

Measures of need for therapeutic security (the DUNDRUM-1), treatment programme completion (DUNDRUM-3) and recovery (DUNDRUM-4) [22] were assessed by a forensic psychiatry lecturer / higher trainee (MD). These SPJ scales are composed of items rated 0 to 4 where ‘0’ indicates no need for therapeutic security, ‘1’ indicates a need for admission to an open ward or equivalent, ‘2’ for low security, ‘3’ for medium security and ‘4’ for high security. For the DUNDRUM-3 and DUNDRUM-4 ‘4’ indicates no readiness for a move to a less secure place, ‘3’ indicates a move from high to medium security, ‘2’ a move from medium to low security, ‘1’ a move from low security to open conditions and ‘0’ indicates no need for therapeutic security. The DUNDRUM-1 (eleven items rated 0 to 44) was used to provide a benchmark for comparative purposes so that other researchers replicating this study or carrying out meta-analyses can compare groups of patients according to their assessed need for therapeutic security. The scale includes items for seriousness of violence and self harm, immediacy of risk of violence and self harm, specialist forensic need, absconding, preventing access to contraband, victim sensitivity and public confidence, complex risk of violence, institutional behaviour and legal process. The DUNDRUM-3 (seven items rated 0 to 28) is a measure of programme completion in domains relevant to risk and harm reduction such as physical and mental health, substance misuse, problem behaviours, self-care and activities of daily living, education, occupation and creativity and family and social networks. The DUNDRUM-4 recovery items (six items rated 0 to 24) include stability, insight, therapeutic rapport, leave, dynamic risk and victim sensitivity.

### Validity of measures

We first measured inter-rater reliability – although this was not necessary in the design of this study. Inter-rater reliability refers to the extent of convergence of judgements about individual items and overall scale scores of different assessors using the tool on the same patient.

We tested concurrent validity with the HCR-20 and S-RAMM because of the expected inverse relationship on the one hand between risk assessment scales HCR-20 and S-RAMM and the protective scales START-strength and SAPROF. The S-RAMM had been validated for the prediction of self-harm in this population and was known to overlap with assessment of risk of violence [32,33]. We also expected positive correlations of HCR-20 and S-RAMM with the START-vulnerability score. We examined concurrent validity with the PANSS because of the known relationship between active symptoms and risk assessment measures [30]. We examined concurrent validity with the GAF because of the expected positive correlation with the protective scales START-strengths and SAPROF and because of the expected inverse relationship with the START-vulnerability scale. Finally we examined concurrent validity with the DUNDRUM-3 programme completion and DUNDRUM-4 recovery scales because they are measures of progress in treatments relevant to risk and increasing strength in domains related to recovery for forensic patients. Lower scores on these scales could be taken to represent ‘negative predictors’ or protective factors.

### Outcome measures

The outcome measures were any adverse events. An adverse event was defined as in the START [19] handbook (page 9) where violence is defined as “any actual, attempted or threatened harm to self or others”. However in this study we have distinguished between violence and self-harm. The START handbook goes on to define self harm as “behaviours involving intentional injury of one’s own body without apparent suicide intent”. We have supplemented this by including any self-harming act whether it was thought to be with suicidal intent or not.

Adverse events were collated by one researcher (ZA) from routine incident report forms. These were supplemented by nurse management daily logs and statutory forms for seclusion and restraint over a 6 month period from March to April 2010 until 31st November 2010. These alternative sources of information acted as a cross check on the completeness of the record of adverse events.

### Study size

All patients in the hospital during the period of baseline data gathering were included. START, SAPROF, HCR-20, S-RAMM, PANSS and GAF were obtained for 98 of 100. The DUNDRUM-1, DUNDRUM-3 and DUNDRUM-4 could not be completed for 6 patients who were discharged before these measures could be completed - they had a significantly shorter length of stay when assessed - 0.28(SD 0.46) years v 7.67(SD 10.09) years, (t = −7.0, df = 98, p < 0.001). The follow-up period was complete to the date patients left the hospital or to the end of the study period.

Based on earlier studies [33,34] we estimated that approximately 10 violent and 10 self-harming adverse events might be expected over a six month period and that these would be sufficient to yield an area under the curve (AUC) in the receiver operating characteristic (ROC) that was capable of being significantly different from the line of random information.

### Quantitative variables

The patients were grouped according to their location in the hospital as this has been established as a proxy for risk levels [23,24,32,33]. Adverse events were further subdivided into violence and self-harm, as outcome measures for the prospective study.

### Statistical methods

All data were analysed in SPSS-20 [34]. Correlations were calculated using the non-parametric Spearman correlation coefficient. Adverse events as outcomes of the prospective study were analysed using the receiver operating characteristic (ROC) area under the curve (AUC). An association was deemed significant if the 95% confidence interval of the AUC was greater than 0.5, the line of random information. The strength of association between measures and outcomes was measured using unadjusted odds ratios (OR). Because the odds ratio is a measure of the increase in odds for each increase of one point in the measurement scale, the magnitude of the odds ration differs according to the properties of item and scale scores. The odds ratio for a scale such as the GAF which is rated 0 to 100 will be inherently smaller when comparing like for like with the HCR-20, a scale rated 0 to 40. Likewise, the odds ratio for items rated 0 to 2 as in the HCR-20, S-RAMM, SAPROF and START will appear larger when comparing like for like with items from the DUNDRUM-1, DUNDRUM-3 and DUNDRUM-4 where items are rated 0 to 4. Confidence intervals for epidemiological rates were calculated using Confidence Interval Analysis [35].

Cronbach’s alpha statistic was used to measure the extent to which each item fits into the subscale or overall scale to which it is allocated. This is a measure of content coherence – whether all items in the overall scale or subscale measure the same thing. High internal consistency also indicates multiple co-linearity for items within a scale.

To examine the extent to which the protective instruments SAPROF, START-S, and recovery instruments DUNDRUM-3 and DUNDRUM-4 were protective in the presence of risk factors, we first carried out an analysis of variance in SPSS-20 with SAPROF, START-S, DUNDRUM-3 and DUNDRUM-4 as dependent variables, violence to others or self-harm as fixed factors (in separate analyses) and the HCR-20-dynamic score as covariate. To examine for interactive effects, we then carried out univariate analysis of variance to examine for main effects and interactive effects.

For item to outcome analysis, the receiver operating characteristic (ROC) area under the curve (AUC) and 95% confidence interval was calculated for each item, with harm to others and self harm as outcome measures. The unadjusted odds ratio (OR) and 95% confidence interval was also calculated for each item and both outcomes, as a measure of the strength of association. Because the items of each scale were strongly inter-correlated, regression models for the items of each scale were not attempted.

## Results

### Participants and descriptive data

The 100 eligible patients included six women and 94 men. Mean age was 40.45 years (SD12.8, range 21.1 to 69.3). The average length of stay at the time of the baseline measures was 7.3 years (SD 9.9, range 0.03 to 44.6 years). Primary diagnosis according to ICD-10 criteria [36] was schizophrenia (ICD-10 F20) 69%, schizoaffective disorder (ICD-10 F25) 16%, bi-polar affective disorder (ICD-10 F31) 7%, recurrent depressive disorder, severe with psychotic symptoms (ICD-10 F33.3) 5%, intellectual disability (moderate mental retardation with significant impairment of behaviour ICD-10 F71.1) 3%.

Mean follow-up time was 181.9 days (SD 70.3, range 0 to 265). The number of patient-days at risk was 18,190.

### Inter-rater reliability of new measures START and SAPROF

For 21 patients rated at different times by SM and ZA, the SAPROF total score correlated Spearman’s r = 0.829, p < 0.001. For the START-strength score, r = 0.694 p < 0.001 and for START-vulnerability score, r = 0.853, p < 0.001. The data subsequently analysed are the ratings made by one researcher ZA. These correlations are given only as an indication of the utility of the instruments.

### Internal consistency

For the SAPROF 17 items Cronbach’s alpha = 0.880, START strengths n = 20 items Cronbach’s alpha = 0.949, START vulnerabilities 20 items Cronbach’s alpha = 0.945, all p < 0.001. No item, if deleted led to a substantial increase in alpha. Internal consistency for these scales is therefore good. Similarly, internal consistency for the PANSS alpha = 0.928 for the full scale, positive sub-scale 0.828, negative sub-scale 0.885, general sub-scale 0.822. The three item supplemental aggression risk (SAR) sub-scale which was not included in the total PANSS score had Cronbach’s alpha = 0.746. The HCR-20 full scale had Cronbach’s alpha = 0.866 with Historical sub-scale 0.672, Current (C) sub-scale 0.843, Risk (R) sub-scale 0.677, dynamic sub-scale (C + R) 0.872. The S-RAMM had a full scale alpha score = 0.672, Background (B) sub-scale 0.485, Current (C) sub-scale = 0.485, Future (F) sub-scale 0.453 and dynamic (C + F) 0.693. For the DUNDRUM-1 triage security scale, Cronbach’s alpha = 0.595, DUNDRUM-3 programme completion scale alpha = 0.912 and DUNDRUM-4 recovery scale alpha = 0.891.

### Construct validity

The SAPROF and START were compared with each other. If the ‘strengths’ scales are valid, they should correlate positively with each other. If the concept of ‘strengths’ is distinct from risks or vulnerabilities, the strengths scales should not correlate strongly with risk or vulnerability scales. The START-S and SAPROF correlated strongly with each other (r = +0.810 p < 0.001), indicating that they measure the same construct. (Table 1). If the START strength and START vulnerability scales measure different constructs, they should not correlate. The correlation between the two was very strong and inverse r-0.947 p < 0.001 indicating that they measure the same thing, one as the inverse of the other.

**Table 1:** Cross validation using Spearman's rank correlation coefficient

	**START-S**	**START-V**	**SAPROF**
SAPROF	+0.810	−0.781	1
START-S	1	−0.947	+0.810
START-V	−0.947	1	−0.781
HCR-H	−0.447	+0.489	−0.394
HCR-C	−0.686	+0.652	−0.775
HCR-R	−0.591	+0.531	−0.670
HCR-dynamic	−0.688	+0.641	−0.779
HCR total	−0.771	+0.701	−0.745
S-RAMM-B	−0.241	+0.288	−0.168
S-RAMM –C	−0.552	+0.503	−0.610
S-RAMM-F	−0.501	+0.477	−0.429
S-RAMM-dynamic	−0.593	+0.554	−0.575
S-RAMM total	−0.546	+0.583	−0.546
GAF	+0.692	−0.644	+0.745
PANSS positive	−0.568	+0.564	−0.620
PANSS negative	−0.573	+0.461	−0.598
PANSS general	−0.513	+0.491	−0.503
PANSS total	−0.606	+0.555	−0.626
PANSS SAR	−0.665	+0.713	−0.564
DUNDRUM-1	−0.373	+0.403	−0.287
DUNDRUM-3	−0.816	+0.769	−0.768
DUNDRUM-4	−0.727	+0.723	−0.680

All significant at p < 0.001 except DUNDRUM-1 vs SAPROF p < 0.005.

### Concurrent validity

The SAPROF and START are said to measure dynamic factors and so they are not expected to correlate with established scales or sub-scales made up of historical or static risk factors. Table 1 shows that the START-S correlated moderately and inversely with HCR-H and weakly and inversely with SRAMM-B. The SAPROF correlated moderately and inversely with the HCR-H and weakly with the S-RAMM-B.

If the SAPROF and START-S measure something different from risk, they should not correlate with the HCR-20 dynamic or S-RAMM dynamic risk assessment scales. Actually they correlated strongly but inversely with the HCR-20 dynamic scale. There was a moderate inverse correlation between the S-RAMM dynamic scale and the START-S. The S-RAMM dynamic score also had a moderate inverse correlation with the SAPROF (Table 1).

START and SAPROF were also compared with measures of global function and mental state. There was a strong positive correlation between GAF and START-S and strong inverse correlation between GAF and START-V. SAPROF and GAF correlated best (Table 1).

Table 1 shows that the PANSS-positive, PANSS-negative, PANSS-general PANSS-total scores and the PANSS-SAR score all correlated strongly and inversely with START-S. PANSS-positive, PANSS-total and PANSS-SAR scores correlated positively with START-V but START-V correlated less well with PANSS-negative and PANSS-general scores (Table 1). The SAPROF correlated inversely with the PANSS scales.

The DUNDRUM-1 correlated weakly and inversely with the START-S, moderately with the START-V and had a weak inverse correlation with the SAPROF. The DUNDRUM3 and DUNDRUM-4 both had strong inverse correlations with the START-S, strong positive correlations with the START-V and strong inverse correlations with the SAPROF.

### Prospective study of violence and self harm

Thirteen individuals had adverse incidents concerning harm to others (broadly defined, as above) during the follow up period and 7 individuals had incidents involving self harm (broadly defined, as above). There was a significant overlap between self-harm and harm to others (X^2^ = 35.2, df = 1, p < 0.001, phi = 0.593, p < 0.001). The rate of events of harm to others (the base rate for violence) was 7.1 per 10,000 patient-days at risk (95% confidence interval 3.8 to 12.2/10,000) and the rate of self-harming events (the base rate for self-harm) was 3.8 per 10,000 patient-days at risk (95% CI 1.5 to 7.9/10,000).

The location at baseline (for eight locations from the most to least secure) predicted harm to others (AUC = 0.812, 95% confidence interval 0.677 to 0.948, p < 0.001) as expected, since we have previously shown that location is a proxy for measures of risk [32] and recovery [23,24]. Length of stay at the beginning of the observation period did not predict harm to others (AUC = 0.504, 95% CI 0.343-0.665, p = 0.963). Location at baseline also predicted self-harm (AUC = 0.838, 95% CI 0.689-0.987, p = 0.003) while length of stay did not predict self harm or the absence of it (AUC = 0.578, 95% CI = 0.383-0.722, p = 0.495).

Table 2 shows that the SAPROF score predicted both the absence of violence and self harm (absence of violence AUC = 0.847 and absence of self-harm AUC = 0.766). The START Strengths and START Vulnerabilities predicted violence (START-S and absence of violence AUC = 0.776, violence; START-V and presence of violence AUC = 0.823) but not self harm.

**Table 2:** Scale scores at baseline and subsequent violence and self harm

	**Harm to others**								**Harm to self**							
	AUC	95% CI of AUC		P	OR	95% CI of OR		p	AUC	95% CI of AUC		p	OR	95% CI of OR		p
		lower	upper			lower	upper			lower	upper			lower	upper	
SAPROF*	0.847	0.721	0.974	0.001	0.775	0.676	0.888	0.001	0.766	0.600	0.931	0.019	0.848	0.738	0.973	0.019
START-S*	0.776	0.653	0.900	0.001	0.903	0.845	0.964	0.002	0.644	0.454	0.833	0.207	0.957	0.891	1.028	0.227
START-V	0.823	0.707	0.940	0.001	1.139	1.057	1.226	0.001	0.654	0.456	0.853	0.175	1.052	0.978	1.131	0.176
HCR-H	0.836	0.747	0.924	0.001	1.226	1.033	1.454	0.020	0.822	0.683	0.960	0.001	1.178	0.998	1.391	0.053
HCR-C	0.794	0.693	0.951	0.001	1.530	1.186	1.974	0.001	0.814	0.617	1.0	0.001	1.603	1.116	2.303	0.011
HCR-R	0.809	0.693	0.924	0.001	1.712	1.265	2.317	0.001	0.790	0.658	0.921	0.001	1.502	1.077	2.094	0.016
HCR-dynamic	0.802	0.656	0.948	0.001	1.309	1.128	1.519	0.001	0.803	0.625	0.980	0.001	1.279	1.067	1.533	0.008
HCR-20 total	0.872	0.783	0.961	0.001	1.236	1.102	1.386	0.001	0.881	0.789	0.973	0.001	1.201	1.058	1.363	0.005
S-RAMM-B	0.766	0.636	0.895	0.002	1.488	1.154	1.918	0.002	0.655	0.459	0.852	0.172	1.248	0.939	1.659	0.126
S-RAMM-C	0.741	0.610	0.872	0.005	1.403	1.105	1.782	0.005	0.813	0.670	0.957	0.006	1.619	1.163	2.253	0.004
S-RAMM-F	0.669	0.526	0.812	0.05	1.108	0.951	1.290	0.190	0.619	0.451	0.787	0.295	1.075	0.894	1.293	0.444
S-RAMM dynamic	0.729	0.593	0.866	0.008	1.136	1.012	1.295	0.031	0.760	0.600	0.921	0.022	1.141	0.998	1.308	0.054
S-RAMM total	0.838	0.756	0.919	0.001	1.196	1.059	1.350	0.004	0.818	0.727	0.909	0.005	1.145	1.014	1.293	0.029
GAF*	0.813	0.651	0.974	0.001	0.909	0.860	0.960	0.001	0.855	0.649	1.0	0.002	0.908	0.850	0.970	0.004
PANSS positive	0.752	0.607	0.898	0.003	1.146	1.050	1.252	0.002	0.764	0.535	0.993	0.02	1.161	1.038	1.299	0.009
PANSS negative	0.639	0.488	0.791	0.106	1.060	0.985	1.141	0.119	0.656	0.472	0.840	0.170	1.057	0.961	1.163	0.256
PANSS general	0.725	0.574	0.877	0.009	1.073	1.016	1.134	0.012	0.823	0.673	0.972	0.005	1.101	1.027	1.180	0.007
PANSS total	0.711	0.577	0.866	0.014	1.035	1.009	1.062	0.009	0.770	0.569	0.971	0.017	1.042	1.008	1.078	0.015
PANSS SAR	0.832	0.730	0.934	0.001	1.509	1.217	1.872	0.001	0.846	0.728	0.965	0.002	1.408	1.118	1.772	0.004
DUNDRUM-1 therapeutic security	0.743	0.603	0.883	0.007	1.238	1.060	1.447	0.007	0.712	0.495	0.929	0.063	1.226	1.012	1.486	0.038
DUNDRUM-3 programme completion	0.832	0.727	0.937	0.001	1.294	1.097	1.525	0.002	0.750	0.596	0.904	0.028	1.184	1.003	1.396	0.046
DUNDRUM-4 recovery	0.728	0.605	0.853	0.011	1.226	1.029	1.461	0.023	0.713	0.557	0.905	0.043	1.227	0.977	1.540	0.079

Area under the curve (AUC) from receiver operating characteristics and unadjusted odds ratios (OR) for 100 in-patients. Six month prospective study, 13 incidents of violence to others, 7 incidents of self harm. Variables marked * (SAPROF, START-S, GAF) have AUC calculated such that smaller test scores indicate higher risk of the adverse events. Unadjusted odds ratios (OR) are given with Wald p values.

By contrast, the HCR-20 predicted both violence (AUC = 0.872) and self harm (AUC = 0.881) as did all of its sub-scales. The S-RAMM predicted violence (AUC = 0.838) though not as quite so well as the HCR-20 and the S-RAMM predicted self-harm (AUC = 0.818) as did the S-RAMM sub-scales, though the S-RAMM-background and future scales did not reach significance for self-harm in this study. It is interesting to note that the SAPROF did almost as well as the S-RAMM as a predictor of the absence of self harm.

The GAF score was a significant predictor of the absence of both violence and self harm, with high AUCs (absence of violence AUC = 0.813 and absence of self-harm AUC = 0.855).

PANSS positive, PANSS general and PANSS total scores each predicted violence and self harm though the PANSS negative symptom score was neither a positive nor a negative predictor for violence or self harm. The odds ratios for these sub-scale scores, though significant, were only modestly better than chance. The PANSS supplemental aggression risk (SAR) score (not included in the PANSS total score) was also a significant predictor of both harm to others and harm to self.

Contrary to expectations, The DUNDRUM-1 triage security score predicted violence (AUC = 0.743) and though the AUC for the prediction of self harm did not reach significance, the odds ratio did (OR = 1.226). The DUNDRUM-3 programme completion score predicted violence (AUC = 0.832) and self-harm (AUC = 0.750), while the DUNDRUM-4 recovery scale also predicted violence (AUC = 0.728) and self-harm (AUC = 0.713).

### Interactive effects between risk factors and protective factors

Univariate analysis of variance was used to test for the presence of interactive effects between risk measures and protective measures.

Tables 3 and 4 show that the SAPROF, START-S, DUNDRUM-3 and DUNDRUM-4 were all significantly different for the 13 patients who were violent when compared to the non-violent. Likewise for the 7 who self harmed compared to those who did not self-harm. However when these results were adjusted for the HCR-20-dynamic score the differences were no longer significant.

**Table 3:** Risk and recovery measures for violent and non-violent individuals compared

	**Crude data-means(SD)**			**Marginal means (SE) adjusted for HCR-20-dynamic**		
	**No violence**	**Violence**	**ANOVA**	**No violence**	**Violence**	
	**N = 85**	**N = 13**	**F/p**			**F/p**
SAPROF	21.8(6.1)	13.1(6.3)	23.3/0.001	21.0(0.5)	18.6(1.4)	2.5/0.1
START-S	25.2(10.4)	14.6(9.1)	12.6/0.001	23.8(0.9)	23.6(2.5)	0.0/0.9
DUNDRUM-3 programme completion	15.0(6.9)	22.5(3.9)	13.4/0.001	15.8(0.6)	17.1(1.7)	0.5/0.5
DUNDRUM-4 recovery	15.4(6.1)	20.0(2.8)	6.6/0.012	16.1(0.5)	15.2(1.4)	0.4/0.5

SAPROF, START-S, DUNDRUM-3 and DUNDRUM-4 scores for those who were violent and non-violent compared, crude data and marginal means adjusted for HCR-20-dynamic scores.

**Table 4:** Risk and recovery measures for self-harmers and others compared

	**Crude data – means(SD)**			**Marginal means (SE) adjusted for HCR-20-dynamic**		
	**No self harm**	**Self harm**	**ANOVA**	**No self harm**	**Self harm**	
	**N = 87**	**N = 7**	**F/p**			**F/p**
SAPROF	21.2(6.5)	14.1(6.4)	6.9/0.01	20.7(0.5)	20.3(1.8)	0.1/0.8
START-S	24.3(10.8)	18.3(10.4)	1.5/0.2	23.5(0.8)	28.3(3.1)	2.3/0.1
DUNDRUM-3 programme completion	15.6(7.0)	21.4(4.5)	4.7/0.032	16.0(0.6)	15.5(2.1)	0.1/0.8
DUNDRUM-4 recovery	15.7(5.9)	21.4(3.5)	3.7/0.055	16.1(0.5)	15.2(1.8)	0.2/0.6

SAPROF, START-S, DUNDRUM-3 and DUNDRUM-4 scores for those who self-harmed and those who did not self harm compared, crude data and marginal means adjusted for HCR-20-dynamic scores.

For harm to others, the SAPROF and HCR-20-dynamic had significant main effects (HCR-20-dynamic F = 3.97, df = 17, p = 0.003, SAPROF F = 4.67, df = 25, p < 0.001) and a significant interaction effect (F = 2.973, df = 38, p = 0.008) indicating that the SAPROF had a ‘true’ protective effect. The SRAMM-dynamic score also had a significant interaction with the HCR-20-dynamic score (HCR-20-dynamic F = 3.828, df = 18, p = 0.001, SRAMM-dynamic F = 3.909. df = 20, p < 0.001, interaction F = 2.794, df = 33, p = 0.003) apparently indicating a synergistic effect. The START-S, DUNDRUM-1, DUNDRUM-3, DUNDRUM-4, PANNS positive, PANSS negative, PANSS general and GAF scores did not have significant interactions with the HCR-20-dynamic score.

The SAPROF did not have significant interactive effects with the START-S, DUNDRUM-1, DUNDRUM-3, DUNDRUM-4, SRAMM-dynamic, PANSS-positive, PANSS-neg-ative or PANSS-general scores, but had a marginal interactive effect with the GAF (main effects SAPROF F = 4.05, df = 25, p < 0.001, GAF F = 5.78, df = 21, p < 0.001, interactive effect F = 1.98, df = 33, p = 0.059).

### Item to outcome analysis

Table 5 shows the performance of each item of the SAPROF as predictors of violence and self-harm. Twelve of the 17 items predicted the absence of violence including factors such as empathy (OR = 0.231), coping ability (OR = 0.187), self-control (OR = 0.205), work and leisure activities (OR = 0.336), financial management (OR = 0.231), motivation for treatment (OR = 0.340) and attitudes towards authority (OR = 0.264). Five items in the SAPROF predicted the absence of self-harm including empathy (OR = 0.293), coping (OR = 0.192), self-control (OR = 0.260), leisure activities (OR = 0.203) and use of medication (OR = 0.314). Odds ratios could not be calculated for items 14 to 17 (intimate relationships, professional care, living circumstances, external control) because for in-patients in a secure hospital there was too little variation in these item scores.

**Table 5:** SAPROF items related to outcomes

	**Harm to others 13/100**								**Harm to self 7/100**							
**SAPROF**	**AUC**	**95% CI**		**P**	**OR**	**95% CI**		**p**	**AUC**	**95% CI**		**P**	**OR**	**95% CI**		**p**
ITEMS		lower	upper			lower	upper			lower	Upper			lower	upper	
SO1 intelligence	.681	.501	.860	.037	0.230	0.071	0.744	0.014	.620	.410	.830	.291	0.392	0.070	1.709	0.213
SO2 secure attachment in childhood	.590	.407	.773	.297	0.473	0.227	0.984	0.045	.571	.330	.813	.530	0.547	0.214	1.399	0.208
SO3 empathy	.757	.618	.896	.003	0.231	0.084	0.630	0.004	.723	.514	.932	.050	0.293	0.084	1.025	0.055
SO4 coping	.743	.614	.873	.005	0.187	0.059	0.593	0.004	.737	.570	.904	.037	0.192	0.041	0.910	0.038
SO5 self-control	.777	.642	.912	.001	0.205	0.078	0.536	0.001	.757	.613	.900	.024	0.260	0.079	0.853	0.026
SO6 work	.700	.565	.834	.021	0.336	0.135	0.841	0.020	.648	.451	.845	.195	0.476	0.164	1.369	0.168
SO7 leisure activities	.786	.659	.914	.001	0.194	0.069	0.542	0.002	.778	.645	.911	.015	0.203	0.051	0.813	0.024
SO8 financial management	.781	.637	.925	.001	0.231	0.093	0.575	0.002	.557	.317	.796	.619	0.231	0.093	0.575	0.002
SO9 motivation for treatment	.710	.541	.879	.015	0.340	0.149	0.775	0.010	.682	.443	.921	.110	0.394	0.137	1.129	0.083
SO10 attitudes towards authority	.749	.587	.911	.004	0.264	0.117	0.596	0.001	.697	.468	.926	.083	0.359	0.113	0.969	0.043
SO11 life goals	.670	.525	.814	.050	0.378	0.141	1.010	0.052	.618	.407	.828	.301	0.551	0.117	1.713	0.303
SO12 medication	.760	.612	.908	.003	0.255	0.102	0.635	0.003	.729	.536	.922	.044	0.314	0.100	0.982	0.046
SO13 social network	.690	.535	.845	.028	0.370	0.160	0.858	0.021	.623	.432	.814	.279	0.560	0.199	1.579	0.273
SO14 intimate relationship	.535	.375	.696	.683					.533	.322	.744	.772				
SO15 professional care	.500	.331	.669	1.000					.500	.277	.723	1.000				
SO16 living circumstances	.494	.326	.662	.946					.495	.274	.715	.961				
SO17 external control	.494	.326	.662	.946					.495	.274	.715	.961				

Area under the curve (AUC) from receiver operating characteristics and unadjusted odds ratios (OR) for 100 in-patients. Six month prospective study, 13 incidents of violence to others, 7 incidents of self harm. Unadjusted odds ratios (OR) are given with Wald p values. Odds Ratios could not be calculated for items SO14 to SO17 as all scored the same way.

Table 6 shows that 16 of the 20 START-strengths items predicted the absence of violence (strongest odds ratios impulse control OR = 0.244, external trigger OR = 0.249), though only one (mental state OR = 0.180) appeared to predict absence of self-harm. Table 7 shows that for START-vulnerabilities, 16 of 20 items predicted violence, not always the same items as for START-strengths (strongest associations ‘relationships’ OR = 5.5, ‘external triggers’ OR = 6.3, ‘conduct’ OR = 5.1), while ‘mental state’ was again the only item predicting self-harm (OR = 3.9).

**Table 6:** START strengths items related to outcomes

**START-S**	**Harm to others 13/100**								**Harm to self 7/100**							
ITEMS	AUC	95% CI		P	OR	95% CI		p	AUC	95% CI		P	OR	95% CI		p
STS1 social skills	.665	.520	.810	.056	0.496	0.230	1.070	0.074	.484	.270	.698	.890	1.152	0.413	3.211	0.786
STS2 relationships	.685	.539	.830	.033	0.395	0.165	0.947	0.037	.496	.274	.718	.972	1.056	0.388	2.872	0.915
STS3 occupational	.720	.569	.871	.011	0.340	0.149	0.775	0.010	.649	.458	.840	.190	0.515	0.193	1.378	0.186
STS4 recreational	.729	.619	.838	.008	0.331	0.142	0.773	0.011	0.599	.424	.773	.385	0.669	0.241	1.855	0.440
STS5 self-care	.713	.565	.860	.014	0.350	0.149	0.821	0.016	.669	.463	.874	.138	0.445	0.152	1.302	0.139
STS6 mental state	.692	.530	.855	.026	0.365	0.151	0.884	0.026	.776	.631	.922	.015	0.180	0.040	0.803	0.025
STS7 emotional state	.622	.468	.776	.157	0.586	0.273	1.255	0.169	.500	.284	.716	1.000	1.031	0.367	2.900	0.954
STS8 substance use	.667	.510	.824	.053	0.506	0.254	1.007	0.052	.568	.360	.775	.553	0.816	0.323	2.061	0.667
STS9 impulse control	.798	.664	.932	.001	0.244	0.111	0.538	0.001	.712	.495	.929	.063	0.375	0.148	0.948	0.038
STS10 external triggers	.753	.600	.906	.003	0.249	0.097	0.642	0.004	.672	.472	.872	.131	0.428	0.149	1.235	0.117
STS11 social support	.625	.496	.754	.148	0.567	0.241	1.335	0.194	.542	.326	.757	.715	0.817	0.267	2.506	0.817
STS12 material resources	.653	.494	.812	.077	0.499	0.237	1.049	0.067	.590	.379	.802	.428	0.679	0.262	1.762	0.427
STS13 attitudes	.736	.609*	.863	.006*	0.337	0.151	0.750	0.008	.619	.443	.794	.298	0.630	0.251	1.586	0.327
STS14 medication adherence	.690	.523*	.857	.028*	0.560	0.224	1.401	0.216	.645	.441	.849	.202	0.410	0.199	0.844	0.016
STS15 rule adherence	.752	.596*	.907	.004*	0.269	0.120	0.603	0.001	.602	.369	.835	.370	0.590	0.224	1.556	0.286
STS16 conduct	.737	.592*	.882	.006*	0.325	0.146	0.723	0.006	.535	.306	.763	.762	0.866	0.336	2.229	0.765
STS17 insight	.656	.506*	.806	.071	0.447	0.183	1.089	0.076	.547	.337	.757	.679	0.808	0.291	2.244	0.683
STS18 plans	.624	.479	.769	.151	0.526	0.236	1.172	0.116	.621	.453	.789	.288	0.509	0.171	1.520	0.226
STS19 coping	.662	.510*	.815	.060	0.407	0.161	1.032	0.058	.502	.260	.743	.989	1.034	0.369	2.892	0.950
STS20 treatability	.724	.606*	.841	.010*	0.319	0.132	0.772	0.011	.619	.469	.768	.298	0.584	0.214	1.588	0.292

**Table 7:** START vulnerabilities items related to outcomes

**START**	**Harm to others 13/100**								**Harm to self 7/100**							
**Vulnerabilities**	**AUC**	**95% CI**		**P**	**OR**	**95% CI**		**p**	**AUC**	**95% CI**		**p**	**OR**	**95% CI**		**p**
Items		Lower	upper			lower	upper			lower	upper			lower	upper	
STV1 social skills	.759	.621*	.897	.003*	4.319	1.592	11.714	0.004	.617	.371	.864	.303	1.760	0.621	4.993	0.288
STV2 relationships	.770	.639*	.901	.002*	5.478	1.685	17.815	0.005	.680	.474	.886	.113	2.575	0.791	8.375	0.116
STV3 occupational	.686	.525*	.847	.031*	2.378	1.126	5.023	0.023	.525	.293	.756	.827	1.131	0.476	2.687	0.780
STV4 recreational	.731	.592*	.871	.007*	3.550	1.403	8.982	0.007	.568	.333	.802	.553	1.391	0.509	3.801	0.519
STV5 self-care	.712	.560*	.863	.014*	2.819	1.287	6.177	0.010	.600	.366	.834	.379	1.732	0.643	4.662	0.277
STV6 mental state	.648	.489	.808	.085	2.092	0.921	4.749	0.078	.728	.577	.880	.045	3.949	0.967	16.129	0.056
STV7 emotional state	.733	.597	.870	.007	3.457	1.428	8.365	0.006	.547	.328	.765	.682	1.263	0.469	3.397	0.644
STV8 substance use	.677	.519	.835	.040	2.232	1.094	4.553	0.027	.576	.366	.786	.504	1.334	0.516	3.447	0.552
STV9 impulse control	.795	.657	.933	.001	4.554	1.976	10.495	0.001	.708	.485	.931	.067	2.836	1.078	7.466	0.035
STV10 external triggers	.784	.675	.893	.001	6.337	1.890	21.243	0.003	.690	.530	.851	.094	2.837	0.847	9.499	0.091
STV11 social support	.699	.551	.848	.021	3.116	1.206	8.048	0.019	.530	.329	.730	.795	1.175	0.416	3.322	0.761
STV12 material resources	.645	.487	.804	.092	2.044	0.931	4.489	0.075	.435	.212	.658	.566	0.714	0.233	2.189	0.555
STV13 attitudes	.783	.662	.903	.001	4.103	1.705	9.875	0.002	.651	.429	.872	.185	1.992	0.790	5.034	0.144
STV14 medication adherence	.613	.447	.778	.192	1.682	0.807	3.505	0.165	.533	.340	.725	.774	0.992	0.350	2.809	0.987
STV15 rule adherence	.764	.625	.904	.002	3.799	1.671	8.637	0.001	.644	.434	.853	.207	1.992	0.732	5.051	0.185
STV16 conduct	.811	.664	.957	.001	5.123	2.332	11.253	0.001	.671	.440	.902	.133	2.349	0.976	5.655	0.057
STV17 insight	.641	.484	.797	.103	2.185	0.860	5.551	0.100	.528	.309	.746	.806	1.147	0.392	3.347	0.805
STV18 plans	.687	.557	.817	.030	3.002	1.151	7.830	0.025	.599	.425	.772	.386	1.765	0.596	5.231	0.305
STV19 coping	.686	.546	.826	.031	2.428	0.934	6.311	0.069	.582	.384	.780	.469	1.227	0.409	3.680	0.715
STV20 treatability	.759	.621	.897	.003	2.614	1.103	6.194	0.029	.617	.371	.864	.303	1.481	0.554	3.964	0.434

Table 8 shows that five of the ten historical items of the HCR-20 predicted violence in this in-patient forensic group (strongest associations ‘early maladjustment’ OR = 1.2 and ‘prior supervision failure’ OR = 1.2) while four of the five ‘current’ or ‘C’ items and three of the five ‘risk management’ or ‘R’ items predicted violence (e.g. C5 ‘unresponsiveness to treatment’ OR = 3.9, R4 ‘non-compliance’ OR = 4.8). H1 ‘past violence’ was not a significant predictor in this population as all subjects scored positive.

**Table 8:** HCR-20 items related to outcomes

	**Harm to others 13/98**								**Harm to self 7/98**							
	**AUC**	**95% CI**		**p**	**OR**	**95% CI**		**p**	**AUC**	**95% CI**		**p**	**OR**	**95% CI**		**p**
		**lower**	**upper**			**lower**	**upper**			**lower**	**upper**			**lower**	**upper**	
H1 Previous violence	.467	.291	.643	.705	0.140	0.008	2.381	0.174	.434	.193	.675	.561	0.065	0.004	1.176	0.064
H2 Young age at first violent incident	.627	.459	.796	.140	2.238	0.820	6.105	0.116	.730	.552	.909	.043	5.510	1.096	27.704	0.038
H3 Relationship instability	.648	.511	.785	.086	3.536	0.901	13.872	0.070	.644	.467	.820	.207	5.510	1.096	27.704	0.038
H4 Employment problems	.758	.649	.867	.003	11.005	1.529	79.186	0.017	.713	.558	.867	.061	5.350	0.769	37.219	0.090
H5 Substance misuse problems	.666	.525	.807	.055	3.278	0.909	11.827	0.070	.613	.406	.820	.321	1.856	0.520	6.632	0.341
H6 Major mental illness	.436	.257	.616	.461	0.472	0.112	1.826	0.277	.446	.209	.683	.636	0.547	0.101	2.970	0.485
H7 psychopathy																
H8 Early maladjustment	.714	.576	.852	.013	3.769	1.216	11.683	0.022	.686	.484	.887	.102	2.858	0.734	11.131	0.130
H9 Personality disorder	.643	.464	.822	.098	2.529	1.090	5.855	0.030	.711	.482	.940	.063	3.731	1.226	11.358	0.020
H10 Prior supervision failure	.705	.581	.829	.017	4.787	1.211	18.929	0.026	.635	.458	.812	.235	2.461	0.632	9.578	0.194
C1 Lack of insight	.666	.515	.816	.055	2.737	0.960	7.804	0.060	.716	.559	.872	.058	5.793	0.792	42.383	0.084
C2 Negative attitudes	.772	.639	.905	.002	3.492	1.632	7.468	0.001	.752	.581	.923	.027	2.990	1.145	7.812	0.025
C3 Active symptoms of major mental illness	.650	.491	.810	.081	1.946	0.953	3.976	0.068	.701	.513	.889	.077	2.618	0.898	7.633	0.078
C4 Impulsivity	.744	.566	.921	.005	4.393	2.046	9.425	0.001	.700	.458	.943	.078	3.192	1.280	7.962	0.013
C5 Unresponsiveness to treatment	.745	.588	.902	.005	3.888	1.534	9.851	0.004	.791	.594	.988	.010	6.145	1.403	26.920	0.016
R1 Plans lack feasibility	.687	.534	.840	.030	2.284	1.122	4.650	0.023	.666	.498	.834	.145	1.876	0.757	4.646	0.174
R2 Exposure to destabilisers	.662	.491	.833	.060	3.061	1.169	8.017	0.023	.678	.450	.906	.117	3.477	1.070	11.301	0.038
R3 Lack of personal support	.647	.492	.802	.088	2.095	0.885	4.960	0.093	.560	.353	.767	.598	1.239	0.383	4.008	0.721
R4 Non-compliance with remediation attempts	.760	.610	.909	.003	4.537	1.873	10.992	0.001	.711	.499	.923	.063	3.206	1.126	9.134	0.029
R5 Stress	.740	.595	.886	.005	4.798	1.725	13.347	0.003	.735	.515	.955	.039	4.686	1.266	17.344	0.021

Note that item H7 ‘psychopathy’ was omitted.

For the prediction of self-harm in this group, two of the ten HCR-20 ‘H’ items, four of the five ‘C’ items and one of the five ‘R’ items were better than chance. As before, odds ratios for individual items were an interesting guide to the relative importance of items, with highest odds ratios for items C5 ‘unresponsiveness to treatment’ OR = 6.1, C1 ‘Lack of insight’ OR = 5.8, H 2 ‘young age at first violent incident’ and H3 ‘relationship instability’ both OR = 5.5.

Table 9 shows that for the S-RAMM, one of the nine background or ‘B’ items, two of the eight current or ‘C’ items and one of the five future or ‘F’ items predicted violence, while two of the nine ‘B’ items predicted self-harm (B1 ‘history of deliberate self harm’ OR = 2.5, B3 ‘previous hospitalisation’ OR = 6.4), as did three of the eight ‘C’ items (C3 ‘psychological symptoms’ OR = 3.8, C7 ‘psychosocial stress’ OR = 3.4 and C8 ‘problem solving deficits’ OR = 7.9).

**Table 9:** S-RAMM items related to outcomes

	**Harm to others 13/98**								**Harm to self 7/98**							
	**AUC**	**95% CI**		**p**	**OR**	**95% CI**		**p**	**AUC**	**95% CI**		**p**	**OR**	**95% CI**		**p**
		**lower**	**upper**			**lower**	**upper**			**lower**	**upper**			**lower**	**upper**	
B1 History of deliberate self harm	.724	.585	.863	.009	2.840	1.298	6.212	0.009	.698	.519	.877	.081	2.451	0.905	6.639	0.078
B2 Seriousness of previous suicidality	.576	.403	.750	.375	1.398	0.721	2.710	0.321	.453	.228	.679	.680	0.822	0.332	2.034	0.672
B3 Previous hospitalisation	.649	.496	.801	.085	2.343	0.901	6.090	0.081	.733	.583	.884	.040	6.404	0.873	46.998	0.068
B4 Mental disorder	.442	.264	.621	.502	0.521	0.136	1.998	0.342	.452	.216	.687	.670	0.592	0.106	3.302	0.550
B5 Substance abuse disorder	.625	.473	.776	.148	1.474	0.476	4.567	0.501	.577	.360	.793	.499	2.118	0.798	5.630	0.133
B6 Personality	.639	.461	.817	.106	2.375	1.048	5.383	0.038	.673	.456	.890	.129	2.570	0.897	7.361	0.079
B7 Childhood adversity	.634	.465	.804	.119	1.853	0.854	4.023	0.119	.564	.332	.795	.575	1.312	0.511	3.369	0.572
B8 Suicide in the family	.590	.410	.771	.296	1.980	0.923	4.246	0.079	.496	.271	.722	.973	1.104	0.333	3.659	0.872
B9 Age, Gender and marital status	.445	.269	.620	.522	0.630	0.189	2.104	0.452	.344	.108	.580	.170	-.329	0.092	1.182	0.088
C1 Suicidal ideation, communication and intent	.571	.389	.752	.412	3.876	0.931	16.134	0.063	.637	.388	.886	.229	6.083	1.368	27.045	0.018
C2 Hopelessness	.528	.350	.706	.743	2.399	0.780	7.382	0.127	.604	.354	.855	.358	4.814	1.373	16.875	0.014
C3 Psychological symptoms	.672	.523	.820	.047	2.204	1.030	4.718	0.042	.745	.609	.881	.031	3.766	1.022	13.875	0.046
C4 Treatment adherence	.627	.462	.793	.140	1.942	0.864	4.369	0.108	.626	.388	.864	.268	2.186	0.777	5.149	0.138
C5 Substance use	.500	.331	.669	1.000					.500	.277	.723	1.000				
C6 Psychiatric admission and discharge	.468	.298	.639	.712	0.377	0.045	3.151	0.368	.435	.202	.669	.570	0.196	0.019	2.027	0.171
C7 Psychosocial stress	.640	.488	.791	.105	2.140	0.897	5.107	0.086	.720	.572	.869	.053	3.360	1.016	11.119	0.047
C8 Problem solving deficits	.739	.616	.862	.006	5.185	1.519	17.693	0.009	.761	.617	.905	.022	7.883	1.039	59.784	0.046
F1 Access to preferred method of suicide	.643	.474	.813	.097	2.456	0.982	6.141	0.055	.588	.350	.825	.441	2.019	0.625	6.518	0.240
F2 Future service contact	.574	.412	.737	.389	1.394	0.684	2.843	0.360	.539	.318	.760	.730	1.188	0.473	2.988	0.714
F3 Future response to drug treatment	.648	.502	.794	.086	1.035	0.835	1.282	0.756	.678	.515	.841	.117	1.046	0.814	1.344	0.726
F4 Future response to psychological intervention	.634	.495	.772	.122	2.276	0.890	5.823	0.086	.606	.424	.788	.351	1.927	0.586	6.338	0.280
F5 Future Stress	.580	.424	.736	.356	2.079	0.587	7.367	0.257	.472	.254	.691	.808	0.911	0.242	3.431	0.890

Note that and odds ratio could not be calculated for C5 ‘substance misuse’ as no subjects scored ‘0’.

Table 10 shows that three of the eleven DUNDRUM-1 triage security items (scored 0 to 4) predicted violence (TS4 ‘immediacy of risk of suicide or self harm’ OR = 1.5, TS9 ‘complex risk of violence’ OR = 3.3, TS10 ‘institutional behaviour’ OR = 2.7) and 3 items predicted self-harm (TS2 ‘seriousness of self harm’ OR = 1.4, TS4 ‘immediacy of risk of suicide/self harm’ OR = 1.5, TS10 ‘institutional behaviour’ OR = 2.7). For the DUNDRUM-3 programme completion items, all seven predicted violence (odds ratios ranged from 1.9 to 4.9) and four predicted self-harm (odds ratios ranged from 1.9 to 4.9). Item PC2 ‘Mental health’ had the strongest odds ratio for both harm to others (OR = 4.9) and to self (OR = 5.2). For the DUNDRUM-4 recovery scale, four of the six items predicted violence and 3 predicted self-harm. Odds ratios were similar for harm to others and harm to self, except for the item derived from the HCR-20 dynamic risk scale, which had a stronger odds ratio for harm to others.

**Table 10:** DUNDRUM-1 triage security items, DUNDRUM-3 programme completion items and DUNDRUM-4 recovery items related to outcomes

	**Harm to others 12/94**								**Harm to self 7/94**							
	**AUC**	**95% CI**		**p**	**OR**	**95% CI**		**p**	**AUC**	**95% CI**		**p**	**OR**	**95% CI**		**P**
		**lower**	**upper**			**lower**	**upper**			**lower**	**upper**			**lower**	**upper**	
	DUNDRUM-1 triage security															
TS1 Seriousness of violence	.454	.275	.634	.610	1.012	0.436	2.347	0.978	.365	.133	.596	.235	0.769	0.344	1.721	0.523
TS2 Seriousness of self-harm	.627	.477	.776	.158	1.278	0.858	1.904	0.227	.665	.506	.824	.148	1.373	0.832	2.265	0.215
TS3 Immediacy of risk of violence	.587	.385	.790	.330	1.517	0.969	2.376	0.069	.701	.459	.944	.078	1.646	0.931	2.909	0.087
TS4 Immediacy of risk of suicide/ self harm	.665	.505	.824	.066	1.545	0.870	2.744	0.138	.704	.528	.881	.073	1.506	0.727	3.121	0.270
TS5 Specialist forensic need	.630	.491	.769	.147	1.239	0.520	2.950	0.629	.623	.459	.788	.280	0.734	0.287	1.879	0.519
TS6 Absconding / eloping	.586	.397	.775	.336	1.239	0.520	2.950	0.629	.499	.235	.764	.994	0.734	0.287	1.879	0.519
TS7 Preventing access	.640	.484	.797	.118	1.926	0.888	4.179	0.097	.526	.321	.732	.818	1.162	0.462	2.923	0.750
TS8 Victim sensitivity/public confidence issues	.504	.280	.727	.968	1.265	0.394	4.060	0.693	.596	.307	.886	.400	1.265	0.394	4.060	0.693
TS9 Complex Risk of Violence	.686	.539	.833	.038	3.324	1.154	9.576	0.026	.644	.455	.832	.208	2.464	0.691	8.781	0.164
TS10 Institutional behaviour	.793	.645	.940	.001	2.665	1.158	6.134	0.021	.787	.662	.912	.012	2.665	1.158	6.134	0.021
TS11 Legal process	.454	.272	.636	.610	2.757	1.427	5.324	0.003	.438	.203	.672	.584	0.479	0.084	2.723	0.407
	DUNDRUM-3 programme completion															
PC1 Physical health	.692	.522	.861	.033	2.078	1.066	4.050	0.032	.617	.383	.850	.307	1.529	0.717	3.258	0.272
PC2 Mental health	.803	.697	.909	.001	4.898	1.646	14.576	0.004	.801	.672	.931	.008	5.175	1.192	22.467	0.028
PC3 Drugs and Alcohol	.771	.619	.923	.002	2.469	1.252	4.871	0.009	.663	.442	.885	.152	1.566	0.808	3.033	0.184
PC4 Problem behaviours	.662	.536	.787	.072	2.041	1.004	4.148	0.049	.677	.525	.830	.120	2.212	0.821	5.958	0.116
PC5 Self-care and activities of daily living	.752	.618	.886	.005	2.978	1.378	6.437	0.006	.705	.502	.909	.072	2.203	0.934	5.197	0.071
PC6 Education, Occupation and Creativity	.817	.682	.951	.000	3.708	1.733	7.936	0.001	.775	.637	.913	.016	2.588	1.145	5.849	0.022
PC7 Family and Social Networks	.698	.544	.852	.027	1.906	1.073	3.386	0.028	.620	.402	.837	.293	1.456	0.750	2.827	0.267
	DUNDRUM-4 recovery															
RC1 Stability	.765	.642	.889	.003	2.493	1.263	4.923	0.008	.760	.619	.902	.022	2.627	1.034	6.674	0.042
RC2 Insight	.649	.511	.788	.096	1.614	0.921	2.828	0.095	.622	.464	.779	.287	1.456	0.728	2.913	0.289
RC3 Rapport and Working Alliance	.676	.535	.817	.050	2.053	1.014	4.157	0.046	.670	.527	.813	.136	2.075	0.832	5.180	0.118
RC4 Leave	.623	.481	.765	.170	1.600	0.890	2.876	0.117	.594	.398	.789	.412	1.406	0.690	2.863	0.348
RC5 HCR-20 Dynamic Items	.671	.539	.803	.057	3.891	0.736	20.571	0.110	.695	.546	.845	.087	1.109	1.029	1.198	0.043
RC6 Victim Sensitivity Issues	.657	.478	.835	.081	1.500	0.886	2.540	0.132	.695	.461	.928	.088	1.666	0.804	3.451	0.170

For the PANSS, Table 11 shows that of the seven positive symptoms (scored 0 to 7), four were significantly associated with violence (‘conceptual disorganisation’ OR = 1.9, ‘hyperactivity’ OR = 3.6, ‘suspiciousness’ OR = 1.5 and ‘hostility’ OR = 2.2). Some of these were marginal (AUC > 0.5) and neither delusions nor hallucinations were associated with violence in this treated in-patient group of patients with severe mental illness, because of lack of variation in the population studied. Of the seven negative symptoms only one, ‘poor rapport’ (OR = 1.7) was associated with violence and of the 16 general symptoms, ‘tension’ (OR = 2.1), ‘uncooperativeness’ (OR = 1.7), ‘poor attention’ (OR = 2.3) and ‘poor impulse control’ (OR = 2.4) were associated with violence. Odds ratios could not be calculated for item G10 ‘disorientation’ as all subjects scored negative.

**Table 11:** PANSS items and AUC for harm to others and harm to self

	**Harm to others n = 13/100**								**Harm to self n = 7/100**							
	**AUC**	**95% CI of AUC**		**p**	**OR**	**95% CI of OR**		**p**	**AUC**	**95% CI of AUC**		**p**	**OR**	**95% CI of OR**		**P**
		**lower**	**upper**			**lower**	**upper**			**lower**	**upper**			**lower**	**upper**	
P1 delusions	.586	.398	.774	.318	1.201	0.901	1.601	0.211	.690	.443	.938	.094	1.475	0.992	2.194	0.055
P2 conceptual disorganisation	.746	.592	.899	.004	1.918	1.282	2.870	0.002	.805	.625	.985	.007	2.336	1.318	4.139	0.004
P3 hallucinations	.656	.473	.839	.070	1.600	1.144	2.238	0.006	.713	.476	.949	.061	1.774	1.173	2.684	0.007
P4 hyperactivity	.780	.612	.949	.001	3.579	1.914	6.694	0.001	.764	.547	.981	.020	2.814	1.369	5.782	0.005
P5 grandiosity	.503	.334	.672	.971	1.019	0.618	1.682	0.940	.502	.274	.731	.984	1.036	0.540	1.986	0.916
P6 suspiciousness / persecution	.665	.516	.814	.055	1.504	0.981	2.306	0.061	.582	.358	.807	.470	1.243	0.715	2.159	0.441
P7 hostility	.755	.618	.891	.003	2.232	1.313	3.795	0.003	.709	.541	.877	.066	1.767	0.919	3.399	0.088
N1 blunted affect	.525	.346	.703	.774	1.075	0.725	1.592	0.720	.573	.334	.812	.521	1.152	0.690	1.924	0.588
N2 emotional withdrawal	.590	.415	.764	.298	1.240	0.816	1.885	0.313	.631	.394	.869	.248	1.380	0.792	2.402	0.255
N3 poor rapport	.714	.574	.853	.013	1.713	1.111	2.642	0.015	.647	.440	.854	.197	1.372	0.807	2.331	0.243
N4 passive / apathetic, social withdrawal	.534	.376	.693	.689	1.076	0.735	1.575	0.707	.487	.307	.667	.909	0.911	0.532	1.560	0.735
N5 difficulty in abstract thinking	.567	.385	.750	.436	1.171	0.821	1.671	0.383	.637	.434	.840	.229	1.330	0.825	2.144	0.242
N6 lack of spontaneity and flow of conversation	.631	.472	.791	.128	1.371	0.923	2.035	0.118	.666	.477	.854	.145	1.434	0.856	2.403	0.171
N7 stereotyped thinking	.614	.428	.800	.186	1.606	1.001	2.575	0.050	.651	.475	.826	.186	1.421	0.980	2.588	0.251
G1 somatic concern	.482	.321	.643	.838	0.765	0.398	1.470	0.422	.536	.301	.771	.751	1.184	0.702	1.999	0.526
G2 anxiety	.551	.383	.718	.556	1.106	0.645	1.896	0.714	.679	.466	.892	.116	1.511	0.840	2.716	0.168
G3 guilt feelings	.419	.263	.575	.348	0.713	0.387	1.312	0.277	.505	.303	.706	.968	0.933	0.473	1.842	0.842
G4 tension	.698	.519	.876	.022	2.085	1.212	3.589	0.008	.764	.541	.988	.020	2.364	1.222	4.571	0.011
G5 mannerisms and posturing	.544	.372	.716	.608	0.965	0.534	1.742	0.905	.577	.341	.813	.499	1.187	0.676	2.082	0.551
G6 depression	.623	.447	.799	.154	1.640	0.932	2.868	0.086	.574	.354	.793	.517	1.175	0.522	2.642	0.697
G7 motor retardation	.501	.330	.673	.988	1.016	0.535	1.930	0.960	.532	.300	.764	.777	1.155	0.537	2.488	0.712
G8 uncooperativeness	.695	.536	.853	.024	1.654	1.075	2.544	0.022	.660	.466	.853	.160	1.422	0.824	2.456	0.206
G9 unusual thought content	.659	.485	.832	.066	1.427	1.011	2.015	0.043	.710	.470	.951	.064	1.648	1.039	2.614	0.034
G10 disorientation	.477	.314	.640	.790					.478	.264	.693	.850				
G11 poor attention	.782	.646	.917	.001	2.291	1.402	3.741	0.001	.832	.740	.924	.004	2.441	1.270	4.692	0.007
G12 lack of judgement and insight	.627	.470	.784	.141	1.291	0.913	1.826	0.149	.682	.501	.863	.109	1.484	0.916	2.406	0.109
G13 disturbance of volition	.626	.445	.806	.146	1.595	1.020	2.496	0.041	.801	.590	1.000	.008	2.320	1.354	3.978	0.002
G14 poor impulse control	.745	.578	.912	.005	2.377	1.409	4.012	0.001	.727	.514	.939	.046	2.066	1.093	3.905	0.026
G15 preoccupation	.650	.480	.820	.082	1.532	0.991	2.366	0.055	.785	.608	.962	.012	2.212	1.256	3.893	0.006
G16 active social withdrawal	.604	.435	.773	.228	1.397	0.890	2.194	0.146	.620	.429	.810	.292	1.278	0.708	2.308	0.416
S1 anger	0.786	0.619	0.954	0.001	2.613	1.515	4.508	0.001	0.835	0.659	1.000	0.003	3.258	1.501	7.072	0.003
S2 difficulty in delaying gratification	0.781	0.624	0.939	0.002	2.190	1.416	3.387	0.001	0.717	0.516	0.918	0.057	1.568	0.985	2.496	0.058
S3 affective lability	0.695	0.511	0.879	0.029	2.239	1.369	3.663	0.001	0.726	0.496	0.956	0.047	2.101	1.201	3.675	0.009

For self-harm, the positive symptoms ‘conceptual disorganisation’ (OR = 2.3), ‘hyperactivity’ (OR = 2.8) and ‘hostility’ (1.8) were again associated with adverse outcomes. For negative symptoms, none were associated with self-harm. Of the general symptoms, only ‘tension’ (OR = 2.4), ‘poor attention’ (OR = 2.4), ‘lack of judgement and insight’ (OR = 1.5), ‘disturbance of volition’ OR = 2.3), ‘poor impulse control’ (OR = 2.1) and ‘preoccupation’ (OR = 2.2) were associated with self-harm.

The three items of the PANSS supplemental aggression risk scale (SAR) predicted both harm to others (‘anger’ OR = 2.6, ‘difficulty in delaying gratification’ OR = 2.2, ‘affective lability’ OR = 2.2) and ‘harm to self’ (anger OR = 3.3, ‘difficulty delaying gratification’ OR = 1.6, ‘affective lability’ OR = 2.1).

## Discussion

### Main findings

This paper presents validation studies for ‘fourth generation’ risk assessment instruments. We have examined the utility of these instruments for assessing risk and protective factors for both violence and self-harm. We have identified both overlaps and differences in the risk factors that contribute to predictions of risk of violence and self-harm. We have included some methodological approaches intended to facilitate future researchers who might replicate this work or include it in meta-analyses. These include stating the base rates for violence and self-harm and giving the DUNDRUM-1 triage security ratings as a means of benchmarking the background need for therapeutic security. We believe the most important finding is confirmation that true protective effects can be identified. The SAPROF, a protective scale does more than assess the absence of risk – the SAPROF also had an interactive effect with the HCR-20, offsetting risk.

The SAPROF and START achieved satisfactory levels of inter-rater reliability. The SAPROF and START have good internal consistency. The START strengths and START vulnerabilities scores were strongly inversely correlated, suggesting that the START strengths score is simply the risk measure repeated. However there is sufficient difference in content between the START strengths and SAPROF on the one hand and the HCR-20 sub-scales to explain the interactive effect between the HCR-20 and the SAPROF, so that the ‘strengths/protective’ paradigm is not merely the same risk factors in new clothes.

The DUNDRUM toolkit instruments were not designed as risk assessment instruments; they were designed to be complementary to risk assessments. The DUNDRUM-1 was included only as a benchmarking measure to enable future replication and meta-analysis. In spite of this, the DUNDRUM-1 triage security scale predicted violence and some of its items predicted violence and also self harm. This may be because items such as suicidal behaviour, complex needs and institutional behaviour are indicators of the seriousness of the behaviour that follows and such acts are easier to detect. DUNDRUM-3 programme completion and DUNDRUM-4 recovery scales were good predictors of violence, comparable to the HCR-20 sub-scales and total score.

The S-RAMM, an assessment of risk of suicide and self-harm, was a predictor of violence and the S-RAMM dynamic score had a synergistic interaction with the HCR-20-dynamic score. The GAF and PANSS scales (other than the PANSS negative score) also performed well as predictors of violence. Although designed as assessments of risk and protective factors for violence, most scales were also predictive of self-harm. The S-RAMM-C scale performed well but the S-RAMM-B (background or fixed historical risk factors for suicide), S-RAMM-F (‘future’ risk factors for suicide), START-S and START-V were notable for their lack of predictive capability for self harm in this study.

The overlap between risk factors for violence and self-harm, and the need to assess both has been established [32,33,37,38]. An item analysis shows considerable overlap of the content of each of the scales examined here. Of these, the DUNDRUM-3 programme completion items appeared particularly strong predictors of self-harm or the absence of it, perhaps because of an underlying element of positive motivation that is inherent in the way each item is defined and rated. Of greatest relevance is that most scale scores were predictors of both violence and self-harm, though this was often because of different items within each scale. Much of this appears to be contextual. In a group made up of forensic patients admitted to a forensic hospital because of severe mental illness and violence, it is not surprising that items such as the first item of the HCR-20 ‘past violence’ should be poor discriminants for further violence in a group where all score positive. It is important to note that in this context items such as the first S-RAMM item ‘past self-harm’ are such good predictors of violence to others, though not self-harm, while items such as HCR-20 C5 ‘unresponsiveness to treatment’ and S-RAMM item C3 ‘psychological symptoms’ and C8 ‘problem solving deficits’ predicted both harm to others and self-harm.

For violence, only some items in each scale were predictive. The highest AUC results were obtained for lack of progress in treatment programmes such as education, occupation and creativity, a low GAF score, conduct problems, lack of progress in mental health programmes, impulse control, adverse institutional behaviour, leisure activities, external triggers, negative attitudes, poor attention, financial problems, hyperactivity and self-control, relationship problems, stability, empathy and hostility.

For self-harm a different selection of items predicted adverse events with the highest AUC results for the GAF, poor attention, conceptual disorganisation, lack of progress in mental health programmes, disturbance of volition, unresponsiveness to treatment, adverse institutional behaviour, preoccupation, leisure activities, hyperactivity, tension, problem solving deficits, stability, self-control and negative attitudes.

A notable feature emerges when unadjusted odds ratios are compared with AUC results. Scales and items with significant AUC statistics may have better than random sensitivity and specificity, but may still be weak predictors. While this reflects the reality of multiple co-linearity, any argument that a risk assessment scale made up of just a few of the strongest items would be sufficient is at odds with the clinical need to take notice of a much wider range of risk and protective factors when planning care and treatment [7,8] and when making recommendations or decisions regarding discharge [25]. However it is also the case that using structured professional judgement instruments to assess treatment needs would be invalid if many of the items were poor predictors on their own. We believe the poor performance of many scale items should lead to two forms of revision of these scales. The first would be to specify that some items are useful only for certain contexts – such as in-patient settings, out-patient community placements or prisons. The second would be to drop some items or refine their handbook definitions.

### Study limitations

This paper describes the predictive validity of the SAPROF, START, DUNDRUM-3 and DUNDRUM-4, PANSS and GAF - a range of risk assessment, symptom measurement and outcome measurement instruments for a group of forensic in-patients, including only 6% women. The outcomes found here may not generalise to other settings. Factors influencing in-patient violence may not generalise to violence in the community. Similarly, self-harm in hospital may not equate to self-harm in the community. Replication in other populations would be helpful. However we found that length of stay did not predict violence or self harm whereas location along the continuum of care did. This demonstrates that in this hospital milieu, location was determined by risk and need for therapeutic security, not by a simple chronological waiting list or tariff for movement from more secure to less secure locations. The location was therefore a proxy for risk and an indication that placement and milieu were appropriate to individual need, to manage and reduce risk [39-41].

Many items included in validated risk assessment instruments such as the HCR-20 appeared not to be predictive in this study. The item H7 ‘psychopathy’ was omitted in keeping with modern practice and the latest revision of the HCR-20. This study size and length of follow-up may have been insufficient – a longer follow-up period would have generated a higher base rate for violence and self-harm. We have stated the exact base rates for these events to enable future research and meta-analysis to make valid comparisons. Future studies in more acute in-patient populations (acute psychiatric intensive care units) might be expected to observe higher base rates, more incidents of violence and self-harm amongst fewer patients. Such a study might show stronger effects for more risk factors. Similarly, studies of community based samples might yield fewer adverse events and very different results for individual items. However the completeness and reliability of the recording of such events in the community might be less reliable.

There may be other predictive factors not included in the instruments studied here. The PANSS does not include specific items for sadness or hopelessness, though the item ‘psychological symptoms’ in the S-RAMM would include such symptoms.

According to Rutter [27] a proper analysis of protective effects or resilience would have to involve examining for the effects of protective factors and the interactions they might have not just with risk factors but also with adverse life-events and difficulties. We found some evidence for an interactive effect between the SAPROF and the HCR-20 dynamic score. However a further analysis of interactions between items is required. It may be that some ‘strength’ items are protective against some ‘vulnerability’ risk items but not others. Analysing the many possible combinations would require very large numbers to allow correction for multiple testing, an analysis beyond the scope of this study. Including the location in an analysis of variance may go some way towards this. In a forensic hospital with high levels of staff to patient ratios and professional, ‘low expressed emotion’ interactions, provocations may be so limited that even high personal risk factors are less likely to lead to violence or self harm than might occur in the community. And high average levels of positive symptoms and some risk factors may overshadow other risk factors, making powerful risk factors appear to be poor discriminants in that setting [42].

Finally, this prospective study covered a six month observation period, though a shorter observation period may have been more meaningful, at least for symptom ratings. A longer observation period might have improved statistical power with more adverse events emerging, but a longer observation period would also raise the problem of the validity of ratings of dynamic risk items which might have changed in the interim. Much remains to be learned about the time course over which dynamic and some static risk items might change.

### Future validation studies

The definitive study would probably have to screen very large numbers of high-risk patients in order to demonstrate an effect, including interactive effects. Serial assessments to demonstrate change and assess the effect of change would be of interest. Mindful of the criteria suggested by the Risk Management Authority of Scotland for an evidence based risk assessment tool, it may be necessary to replicate studies such as this in different populations and cultures. Future studies should also consider the synergistic interactions between individual items. True ‘protective effects’ may only emerge from such studies [43,44].

### Advantages of fourth generation (START and SAPROF) structured professional judgement instruments in clinical practice

Measures of strengths and protective factors such as START-S and SAPROF, as well as measures of progress in treatment programmes and recovery such as the DUNDRUM-3 and DUNDRUM-4 performed well in this study of in-patient violence and self-harm in a forensic setting. While identifying items as ‘protective’ rather than ‘vulnerability’ factors may to some extent be a matter of semantics (the START-S and START-V appear to be simple reciprocals of each other), new content can be found in the SAPROF and in the DUNDRUM-3 programme completion and DUNDRUM-4 recovery instruments as well as some START items.

The S-RAMM item B1 “history of deliberate self harm” predicted harm to others as well as harm to self (AUC 95% CI greater than 0.5) in this population. In contrast, the HCR-20 H1 item “previous violence” was a poor discriminator in this forensic population, because almost all patients scored positive. This may be an unexpected benefit of using diverse assessments and may also be understandable in psychological terms. The S-RAMM is also valuable because so much of the item content is not replicated in violence risk assessment instruments.

The new content of instruments such as the SAPROF, START, DUNDRUM-3 and DUNDRUM-4 as well as the S-RAMM lends itself to the use of risk assessment as a form of needs assessment and a means for planning care and treatment. These positively connoted factors are more likely to be acceptable to patients or service users when working to engage them in recovery oriented programmes in which risk management is important.

The pairing of assessments of risk of violence to others with assessments of risk of self-harm and suicide is similarly a means of identifying the process of risk assessment and risk management with the patient or service user’s best interests rather than a process intended exclusively to serve the purposes of criminal justice and public protection.

The START has been shown to have good psychometric properties and to be predictive of violence when used as part of the assessment for mental health review boards in a forensic hospital [45]. De Vries Robbé et al. found that the use of the SAPROF can be helpful in formulating treatment goals, progressing through stages of treatment, planning the phasing of treatment and facilitating risk communication [46,47]. The DUNDRUM-3 and DUNDRUM-4 are intended to serve the same processes of treatment planning and measurement of treatment outcome in domains relevant to risk reduction and risk management and to provide transparency when reporting to mental health tribunals and boards [22-26].

The GAF, a simple global assessment of function, performs as well as many of the more specific assessment instruments. It may be that global function is the most consistent underlying measure of risk and resilience. Alternatively this may be an example of the use of an ‘intuition based’ assessment. Carroll [48] has recently pointed out the advantages of such intuitive approaches while cautioning that a structured professional judgement instrument should always be used alongside intuitive assessments as a valid, transparent, deliberative and unbiased check on some of the problems that can arise with intuitive methods.

## Conclusions

The START and SAPROF have good psychometric characteristics for use as clinical or research instruments in severe mental illness. The SAPROF predicted the absence of violence and self harm. The START-Strengths predicted absence of violence but not self harm. The DUNDRUM-3 programme completion and DUNDRUM-4 recovery instruments also predicted violence and self-harm. They were not consistently better than the HCR-20, S-RAMM, PANSS or GAF in predicting adverse events-violence or self harm. The SAPROF, START, DUNDRUM-3 and DUNDRUM-4 however have the advantage of covering a wider content than existing risk assessment instruments and have different purposes. The GAF performs as well as the scale scores of most specific risk assessment instruments. Many individual items in the SPJ instruments studied were strongly associated with adverse outcomes in this setting, meriting further study of context and interactive effects.

## Competing interests

The authors declare that they have no competing interests.

## Authors’ contributions

ZA rated patients using the SAPROF and START and collated the adverse events. MD rated the DUNDRUM-1, DUNDRUM-3 and DUNDRUM-4. LN and OG both rated patients using the PANSS and GAF. AN coordinated and collated ratings of HCR-20 and S-RAMM. HGK wrote the first draft of the paper, designed the study and carried out the data analysis. All authors reviewed the drafts and agreed the final text. All authors read and approved the final manuscript.

## Pre-publication history

The pre-publication history for this paper can be accessed here:

http://www.biomedcentral.com/1471-244X/13/197/prepub
